# An e-Learning Course to Train General Practitioners in Planetary Health: Pilot Intervention Study

**DOI:** 10.2196/56138

**Published:** 2024-05-14

**Authors:** Cédric Tourrette, Jean-Baptiste Tostain, Eva Kozub, Maha Badreddine, Julia James, Aurore Noraz, Charlotte De Choudens, Lionel Moulis, Claire Duflos, Francois Carbonnel

**Affiliations:** 1 University Department of General Practice Montpellier University Montpellier France; 2 Desbrest Institute of Epidemiology and Public Health Montpellier University, INSERM Montpellier France; 3 University Department of General Practice University Toulouse III Toulouse France; 4 Department of Pedagogical Engineering and Audiovisual Production Faculty of Medicine Montpellier University Montpellier France; 5 Clinical research and Epidemiology Unit University of Montpellier Hospital Center Montpellier France; 6 Maison de Santé Pluriprofessionnelle Universitaire Avicenne Cabestany France

**Keywords:** planetary health, One Health, medical education, environmental health, education, e-learning, general practitioner, pilot study, climate change, training, environmental, e-learning module, behavior change, ecosystem, questionnaire, behavior, self-assessment, e-learning intervention, environment

## Abstract

**Background:**

According to the World Health Organization, climate and ecological emergencies are already major threats to human health. Unabated climate change will cause 3.4 million deaths per year by the end of the century, and health-related deaths in the population aged ≥65 years will increase by 1540%. Planetary health (PH) is based on the understanding that human health and human civilization depend on flourishing natural systems and the wise stewardship of those natural systems. Health care systems collectively produce global emissions equivalent to those of the fifth largest country on earth, and they should take steps to reduce their environmental impact. Primary care in France accounts for 23% of greenhouse gas emissions in the health care sector. General practitioners (GPs) have an important role in PH. The course offers first-year GP residents of the Montpellier-Nîmes Faculty of Medicine a blended-learning course on environmental health. An e-learning module on PH, lasting 30 to 45 minutes, has been introduced in this course.

**Objective:**

The objective of this study was to assess the impact of the e-learning module on participants’ knowledge and behavior change.

**Methods:**

This was a before-and-after study. The module consisted of 3 parts: introduction, degradation of ecosystems and health (based on the Intergovernmental Panel on Climate Change report and planetary limits), and ecoresponsibility (based on the Shift Project report on the impact of the health care system on the environment). The questionnaire used Likert scales to self-assess 10 points of knowledge and 5 points of PH-related behavior.

**Results:**

A total of 95 participants completed the pre- and posttest questionnaires (response rate 55%). The mean scores for participants’ pretest knowledge and behaviors were 3.88/5 (SD 0.362) and 3.45/5 (SD 0.705), respectively. There was no statistically significant variation in the results according to age or gender. The pretest mean score of participants who had already taken PH training was statistically better than those who had not taken the PH training before this course (mean 4.05, SD 0.16 vs mean 3.71, SD 0.374; *P*<.001).

**Conclusions:**

The PH module of the Primary Care Environment and Health course significantly improved self-assessment knowledge scores and positively modified PH behaviors among GP residents. Further work is needed to study whether these self-declared behaviors are translated into practice.

## Introduction

According to the World Health Organization, climate and ecological emergency changes will be the main threats to human health over the coming decades [1]. Unabated climate change will cause 3.4 million deaths per year by the end of the century, and health-related deaths in the population aged ≥65 years will increase by 1540% [2]. By 2030, its financial cost to human health is estimated between US $2 and $4 billion per year [1]. According to the Intergovernmental Panel on Climate Change, its consequences are increasing the pressure on health care systems, which are already under strain, and will further penalize the most fragile populations [3]. Health care systems have a significant impact on climate change. Health care systems collectively produce global emissions equivalent to those of the fifth largest country on Earth [4]. In France, this impact is estimated at 8% of the national total [5]. Specifically, primary care in France accounts for 23% of greenhouse gas emissions in the health care sector [5]. The World Organization of General Practitioners has called for the promotion of planetary health (PH) and environmentally responsible practices [6]. PH is based on the understanding that human health and human civilization depend on the flourishing of natural systems and the wise stewardship of these natural systems [7]. General practitioners (GPs) hold the status of a reliable source of information for the public, particularly regarding health and the environment [8]. Most of them express concern and are in favor of changing their practices. However, they often find it difficult to translate this desire into action due to constraints such as lack of time, the complexity of their practice, the weight of established habits, and insufficient training on the subject [9]. It has been known that offering training in PH to medical students leads to a significant increase in their beliefs about the need for climate change education and related physician responsibilities [10]. Intentions to change personal behaviors and apply new knowledge in future clinical practice have also significantly increased. Training health professionals in environmental health (EH) is one of the priorities set by the fourth French national EH Plan [11]. EH is defined as “those aspects of human health, including quality of life, that are determined by the physical, chemical, biological, social, psychosocial, and aesthetic factors in our environment” [12]. The national plan is deployed by regional health agencies, which determine the national priorities most relevant to their region. The Occitanie Health Regional Agency funded the creation of the Primary Care Environment and Health (PCEH) course (known as *SPES* in French for *Soins Primaires Environnement et Santé*) in 2021 to train GP residents from the Montpelier-Nimes Faculty of Medicine in EH. This mandatory training was organized in 2 successive parts: an asynchronous e-learning modular course focusing on EH knowledge and tools, followed by a day of face-to-face sessions. The face-to-face sessions included lectures by EH experts in the morning and problem-solving workshops in the afternoons to support thesis work in EH. The e-learning was designed to serve as a knowledge base that could be reused each year, ensuring the sustainability of the PCEH course. As it successfully improved both the knowledge and behaviors of GP residents in EH, this course has been extended until 2026, with the aim of adding new modules each year [13]. In 2022, a new module was added to the PCEH course, called PH in GP. It responded to a strong demand from medical residents [14]. To date, we have not found any equivalent study among GP residents, particularly in France.

This pilot study aims at assessing the impact of the PH module of the PCEH course on the knowledge and behavior of GP residents of the Montpellier-Nîmes Faculty of Medicine. The results from this pilot study will be used to improve both the existing e-learning content and the study design of the upcoming planned modules. In 2026, the French general medicine curriculum will include an additional year of junior doctor status. Environmental health and PH will be some of the new themes taught during this year. This study is an opportunity to evaluate this training program in preparation for more advanced studies (particularly focusing on the clinical impact) within this junior doctor year.

## Methods

### Design

We conducted an interventional study using a before-and-after, open, nonrandomized, monocentric design. It was reported following guidelines for nonrandomized pilot and feasibility studies [15] and in line with the CONSORT (Consolidated Standards of Reporting Trials) 2010 checklist for reporting a pilot or feasibility trial (items pertinent to randomization were considered not applicable) [16].

To structure our inquiry, we relied on a commonly used framework for evaluating learning, known as the Kirkpatrick model [17]. This model frames training on 4 levels: reaction, learning, behavior, and results. “Reaction” is the degree to which participants find the training favorable, engaging, and relevant to their jobs; “learning” focuses on the degree to which participants acquire the intended knowledge, skills, attitude, confidence, and commitment based on their participation in the training; “behavior” examines the degree to which participants apply what they learned during training when they are back on the job; “results” measures the extent to which targeted outcomes occur as a result of the training and the support and accountability package. A more recent version of the model adds that the behavior level refers to “required drivers” or factors that increase the likelihood that people will retain and apply what they have learned in a given setting [18].

### Participants

The study population included the first-year GP residents at the Montpellier-Nimes Faculty of Medicine who had completed the PH module of the PCEH course. The study design included a preintervention questionnaire, the PCEH course, and the postintervention questionnaire completed immediately after the intervention.

An email was sent to the potential participants, inviting them to identify themselves on Moodle, the learning management system of the University of Montpellier. The average duration of the PH module was estimated to be between 30 and 45 minutes. The questionnaire took between 3 and 5 minutes to complete. Only participants who completed the pre- and posttest questionnaires were included in the final analysis. Students were given 3 months to complete the questionnaires (closing date: January 10, 2023).

### e-Learning Intervention Content

At the beginning of the summer of 2022, a working group was set up by FC, EK, JJ, and CT. It designed the PH module, comprising 3 parts (Table 1).

**Table 1:** e-Learning planetary health (PH) module content.

Modules with intermediate and specific learning objectives			Supports
1. Introduction to the PH module: introduce the issues and the composition of the module			Introductory video
**2. Degradation of ecosystems and health (One Health approach by the WHO^a^: links between human-induced ecosystem degradation and health)**			
	Describe the state of the planet’s health	Text document, illustrated with figures	
	List planetary limits and analyze their impact on health	Text and images	
**3. Ecoresponsibility and environmental health advice: applying the principles of ecoresponsibility and EH^b^ advice (notion of cobenefit) to clinical practice**			
	Describe the impact of the health care system on the environment	Commented slideshow	
	Describe the ecoresponsible approach (identify the cobenefits of an ecoresponsible approach)	Text document, illustrated with figures	

^a^WHO: World Health Organization.

^b^EH: environmental health.

The first part included an introductory video. The second part was based on the Intergovernmental Panel on Climate Change report on climate change and the notion of PH [2]. The third part was based on the Shift Project [4] and the ecoresponsible approach in private practice [19]. The module was then formatted using Rise 360 software (Articulate Global Inc). This software facilitates an interactive layout of the teaching and allows the integration of videos, slideshows, and various text formats. This PH module follows the 3 existing PCEH modules; none of these courses focused on PH (Table 2).

**Table 2:** Three other existing modules of the Primary Care Environment and Health (PCEH) course.

Modules (intermediate learning objectives) and specific learning objectives		Supports
**1. Introduction to EH^a^: raising awareness of the impact of the environment on health**		
	Define EH and the exposome	Commented slideshow
	Define an EH risk	8 videos (outdoor air, indoor environment, water, noise, UV, classified facilities and emitters, endocrine disrupters, and vector-borne pathologies)
	Describe current regulations on environmental risks	Interactive texts
	Define a territorial EH diagnosis	Text document, illustrated with figures
	Describe the fourth national EH plan	Commented slideshow, and when relevant, examples of potential medical thesis topics
**2. Population-based approach: identifying the environmental risks of a population**		
	Differentiate population-based approach, individual approach, and situational analysis	Interactive texts
	Identify an EH risk in an area using mapping tools (Géodes, Atlasanté, and SIRSé^b^)	Interactive texts and videos
	List the EH resources available to GPs^c^	Interactive texts
	List the tools (institutional and noninstitutional) for assessing and analyzing the various risks associated with an area and a population (Occitanie territorial EH diagnosis)	Interactive texts and commented slideshows
	Organize monitoring methods for the practice’s patients (Recosante, Atmo France^d^, Pollens.fr, and DGS^e^-urgent)	Interactive texts
	Describe how to report an EH risk event to health authorities	Interactive texts
3. Integrating EH into my practice for my patients: identify the environmental risks to which an individual is exposed in a clinical case, use tools to assess the various risks associated with an area and a population in an educational situation, and include the patient’s opinion in defining an appropriate response to their EH risk.		A total of 3 clinical cases based on 5 to 6 multiple-choice questions (exposure to lead, atmospheric pollution, and ultraviolet radiation), immediate debriefing of the answers to help participants realize how far they had progressed, and the practical usefulness of the tools proposed

^a^EH: environmental health.

^b^SIRSé: Système d’information regional en Santé.

^c^GPs: general practitioners.

^d^Atmo France: French Federation of Air Quality Monitoring Associations (Fédération des Associations Agrées de Surveillance de la Qualité de l'Air).

^e^DGS: Direction Générale de la Santé.

### Data Collection

**Figure 1:**
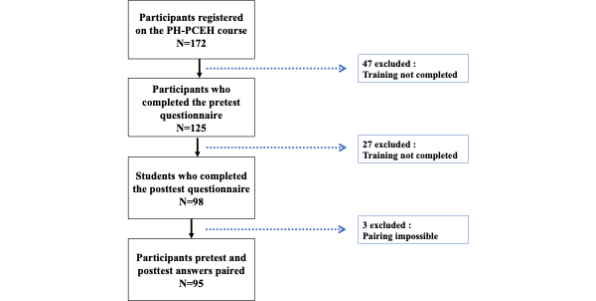
The study flowchart. PH-PCEH: Planteray Health-Primary Care Environment and Health.

A literature search was carried out before starting this study to identify a validated PH training evaluation questionnaire suitable for the study population. However, they were judged to be unsuitable for the course objectives or the population, and a new questionnaire was designed [10,20,21]. These questionnaires were created by the author team due to the absence of an existing validated questionnaire for this population within this study context. It consisted of 20 questions organized into 3 parts (Multimedia Appendix 1). These parts were as follows:

- 5 questions to identify the characteristics of the sample: gender, age, previous university course on PH, concerns about climate change, and feeling to be currently acting in an ecoresponsible way.
- A 10-question section using Likert scales ranging from a minimum of 1 (indicating “strongly disagree”) to a maximum of 5 (indicating “strongly agree”) to assess PH knowledge (level 2 of the Kirkpatrick’s model; Figure 1)
- A 5-question section using Likert scales to describe self-declared PH behaviors as defined by the more recent version of the Kirkpatrick model [18], including the appropriation of knowledge and level of confidence in their abilities at the end of the course, which are markers of behavior changes (level 3 of the Kirkpatrick’s model).

The questionnaires were anonymized by generating a unique identification number on Moodle to match the pre- and posttest results.

A prototype questionnaire was tested and refined through exchanges with authors. The data were extracted by the educational engineer and then transposed into usable numerical data on a Microsoft Excel 2007 spreadsheet.

### Data Analysis

The quantitative variables were presented using mean (SD) values. Differences in pre- and postintervention Likert scales were also presented as mean (SD) values, and Wilcoxon signed rank tests were used to analyze these differences.

The pretraining Likert scales were divided into 3 qualitative variables: rather disagree, not at all agree (scores 1-2), neither agree nor disagree (score 3), rather agree or strongly agree (scores 4-5). These were described by their numbers and percentages.

A multivariate analysis was conducted to analyze the relationship between response means and gender or age using the Belsley-Kuh-Welsch technique, after checking that the explanatory variables were not collinear. The variations between the means of the pretraining responses and the difference of the pre- and posttraining means according to the type of previous training in PH were analyzed using the student *t* test (unpaired) after checking the conditions of its application.

The correlation between the means of the pretraining responses and the change in means was compared using Pearson coefficients.

These analyses were conducted by LM, a methodologist, and CDC, a statistician, from the clinical and epidemiological research unit at Montpellier University Hospital. They were carried out using 2-tailed tests with alpha at 5%, using SAS 9.14 and EasyMedStat 3.25 software.

### Ethical Considerations

The need for consent was deemed unnecessary in accordance with national regulations. In France, research on changes in practices resulting from training medical or paramedical staff for research purposes is considered noninterventional, and therefore, does not require an ethical opinion [22].

Ethics committee approval was not required for this study according to General Data Protection Regulation recommendations. All data for this study are stored on a hard disk protected by secure authentication. Participant data were anonymized. Participants were not compensated to take part in this program, which was valued as part of their general medicine curriculum.

## Results

### Participants

The study was carried out from October 13, 2022, to January 10, 2023.

Of the 172 participants registered for the PH-PCEH course, 95 completed the pre- and posttest questionnaires (response rate 55%; Figure 1). The population description is presented in Table 3. The CONSORT 2010 checklist of information to include when reporting a pilot or feasibility trial is available in Multimedia Appendix 2.

**Table 3:** Population description.

Sociodemographic characteristics			Values, n (%)
**Gender**			
	Female	63 (66,3)	
	Male	32 (33.7)	
**Age (years)**			
	20-25	22 (23.2)	
	25-30	67 (70.5)	
	>30	6 (6.3)	
**Statements**			
	I’ve already taken part in a university course on planetary health	8 (8.4)	
	I have concerns about climate change.	92 (96.8)	
	I feel that I am currently acting in an ecoresponsible way.	81 (85.3)	

### Pretest Knowledge and Behaviors

**Figure 2:**
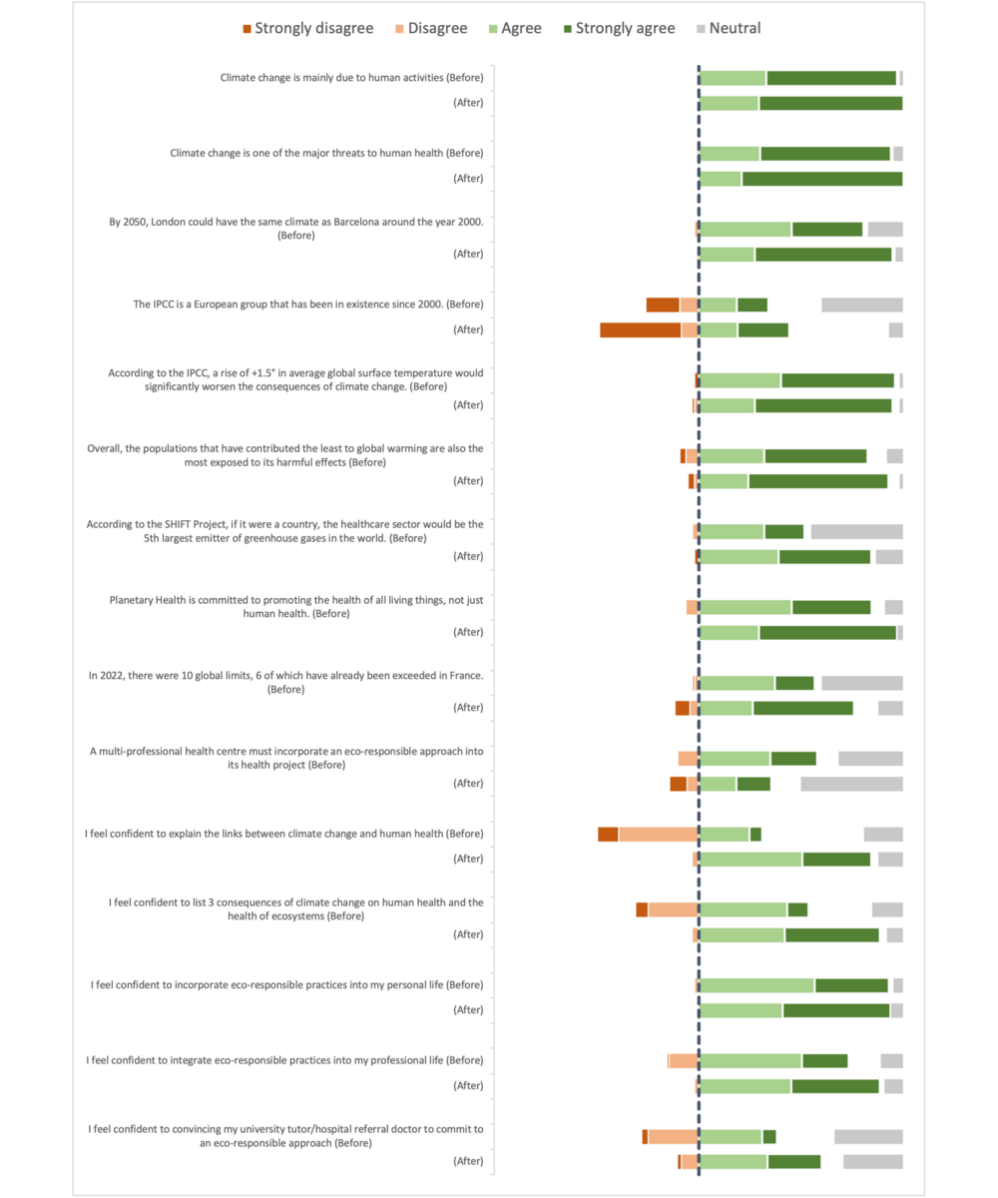
Pre- and postresponses to Likert scale questions on knowledge and behavior.

Pre- and posttest responses to Likert scale questions on knowledge and behavior are presented in Figure 2. The mean scores for participants’ pretest knowledge and behaviors were 3.88/5 (SD 1.12) and 3.45/5 (SD 1.12), respectively. Detailed descriptive statistics for each proposition are shown in Table 4.

There was no statistically significant variation in the results according to age or gender. The pretest mean score of participants who had already taken PH training was statistically better (mean 4.05, SD 0.16 vs mean 3.71, SD 0.374; *P*<.001).

**Table 4:** Pretest knowledge and behavior as well as the pre- and posttest difference in knowledge and behavior (Δ). *P*<.05 (italicized *P* values) are considered significant.

The level based on the Kirpatrick model and questions			Δ (SD)		*P* value
**II: knowledge**					
	Change is mainly due to human activities.	0.12 (0.54)		*.04*	
	Climate change is one of the major threats to human health.	0.23 (0.59)		*<.001*	
	By 2050, London could have the same climate as Barcelona around the year 2000.	0.45 (0.8)		*<.001*	
	The IPCC^a^ is a European group that has been in existence since 2000.	0.25 (1.72)		.14	
	According to the IPCC, a rise of +1.5° C in average global surface temperature would significantly worsen the consequences of climate change.	0.12 (0.73)		.09	
	Overall, the populations that have contributed the least to global warming are also the most exposed to its harmful effects.	0.33 (0.87)		*<.001*	
	According to the Shift Project, if it were a country, the health care sector would be the fifth largest emitter of greenhouse gases in the world.	0.57 (0.95)		*<.001*	
	Planetary health is committed to promoting the health of all living things, not just human health.	0.44 (0.83)		*<.001*	
	In 2022, there were 10 global limits, 6 of which have already been exceeded in France.	0.33 (1.2)		*.01*	
	A multiprofessional health center must incorporate an ecoresponsible approach into its health project.	0.16 (1.2)		.20	
**III: behavior**					
	I feel confident to explain the links between climate change and human health.	1.37 (1.01)		*.001*	
	I feel confident to list 3 consequences of climate change on human health and the health of ecosystems.	1.04 (1.03)		*<.001*	
	I feel confident to incorporate ecoresponsible practices into my personal life.	0.2 (0.61)		*.001*	
	I feel confident to integrate ecoresponsible practices into my professional life.	0.49 (0.91)		*<.001*	
	I feel confident to convince my university tutor or hospital referral doctor to commit to an ecoresponsible approach.	0.58 (0.83)		*<.001*	

^a^IPCC: Intergovernmental Panel on Climate Change.

### Posttest Knowledge and Behaviors

The mean difference for each knowledge and behavioral question is described in Table 4. The mean progression in knowledge and behaviors was 0.35/5 (SD 0.15) and 0.74/5 (SD 0.47), respectively. For all but 3 questions, the postquestionnaire score was statistically higher than the prequestionnaire score.

The medians of the differences for the behaviors were found to be statistically significantly higher than those for the knowledge (median difference –0.3, IQR 0-1; *P*<.001).

The higher the pretraining averages, the lower the changes between pre- and posttraining averages (*r*=–0.41; *P*<.001).

There was no statistically significant variation in posttraining results according to the age or gender of the participants, nor was there an impact observed from previous training in ecoresponsibility or sustainable development.

## Discussion

### Principal Results

The PH module of the PCEH course improved the participants’ knowledge and behaviors. The knowledge level significantly increased on most questions, rising from 3.88 to 4.18 out of 5 (+6%), which is consistent with findings in the existing literature [23,24]. The progress reported in other studies was more substantial, but these studies lack cross-comparability. Their formats vary widely, ranging from about 30-45 minutes asynchronous e-learning module (as in our study) to 75 minutes of supervised telementoring per week over 8 weeks [23], 14 hours of e-learning [24], or 3 live distance learning sessions coupled with 3 blended-learning sequences [25]. We selected our format intending to improve knowledge and promote behavior related to ecoresponsibility and climate change, focusing on its practical and accessible aspects [26]. This approach also has the potential to reshape the carbon footprint of a training course for health care professionals, particularly by reducing the number of journeys required [27]. Fossil fuel transport is indeed one of the main contributors to emissions driving climate change, and some of our participants must drive more than 2 hours to get to the university once a month to follow their course [5]. Climate change is becoming a significant concern among medical students, as stated by 97% of our participants [10]. Training them in this area may be a game changer for the sustainability of the health care system, considering our findings that GP residents with previous PH training achieved better results, a point that has not yet been thoroughly studied. The French Conference of Deans of Medicine has created an e-learning course called “Medicine and Environment,” which has been implemented in medical schools since 2023. It could pave the way for the dissemination of a PH culture in medical training [28].

There is still room for improvement, as 48% of our participants incorrectly assessed the carbon footprint of the health care system by answering “neutral or rather disagree” to the question “according to the Shift Project, if it were a country, the health care sector would be the fifth largest emitter of greenhouse gases in the world”), and 46% did not feel confident in listing 3 impacts of climate change on health. Similar findings have been reported in the United States [28]. We were relieved to note that the understanding of the impact of temperature rise and the human origins of climate change was unanimously shared by our participants, as expected [10]. This is in contrast to the climate skepticism observed in the French population, estimated at 37% [29].

This module led to an improvement in reported behaviors (+14.8%). The ability to explain the links between climate change and health showed the greatest increase (+1.37 points), as expected [10,24]. This aptitude, in turn, can strengthen one’s ability to communicate on the subject [22,30]. Understanding these connections serves as the foundation of this training, aimed at establishing sustainable medicine [31].

In our study, the increase in EH behavior was greater than the increase in knowledge. Combining e-learning with a 1-day face-to-face session may accentuate the improvement in behavior-related scores, as blended-learning is valued by medical students [32].

The reality of behavior change can only be verified at the end of medical studies when these practices are implemented in their future practices. There is considerable room for the sustainability of health care system; most hospitals do not have a greenhouse gas emissions report, although it is compulsory and the ecoresponsibility score of GPs is evaluated at 23/40 [5,33].

### Strengths and Limitations

PH courses are still rare but growing. GP residents with previous PH training showed better results in our study, supporting the integration of a PH culture in medical courses. It was decided not to measure the satisfaction rate, as 94.6% of participants were satisfied with the 3 preexisting modules of the PCEH course [13]. The high completion rate, with a low number of dropouts, suggests that the new module is well accepted. The e-learning format allows residents to follow the module at their own pace. The questionnaire was tailored to correspond to the key points of the training, enabling precise measurement of changes in knowledge while recording changes in self-declared behavior. These changes were statistically significant for most questions. Further work is required to monitor the persistence of these results over time.

Our main limitation was the self-reported nature of behaviors, which may lead to an overestimation of behaviors. A selection bias and social desirability bias may have influenced the responses. Similarly, a halo effect or acquiescence bias cannot be ruled out, which may explain the absence of any significant change in knowledge questions 4 and 10. The fact that the behaviors were self-reported and not observed “on the job” is also questionable. The more recent version of the Kirkpatrick model adds that the behavior level can be seen as catalysts for applying what has been learned; these catalysts include processes and systems, such as job aids, coaching, work review, and incentive systems [18]. Feeling confident in explaining the links between climate change and human health, listing 3 consequences of climate change on human health and the health of the ecosystem, integrating ecoresponsible practices into one’s personal life, integrating ecoresponsible practices into one’s professional life, and convincing the university tutor or hospital referral doctor to commit to an ecoresponsible approach are “required triggers” to change, as described in the Kirkpatrick model [18].

A control group could have increased the reliability of the results and helped to limit possible biases. This could have been achieved by comparing 2 classes from different universities, with one class receiving the Public Health (PH) module and the other acting as the control group. This could be considered in preparation for the junior doctorate year, which will integrate environmental health and PH, as many faculties have not yet designed a PH course for GPs [34].

Finally, a standardized questionnaire on health care professionals' knowledge of PH would have been a valuable evaluation tool. To our knowledge, none currently exists.

### Perspectives

This PH module has proved to be a solid foundation on which to build higher-level assessments (the “results” stage of the Kirkpatrick model). The introduction of the junior doctorate year in GP in 2026 in France will be an opportunity to rethink training during the GP internship to more broadly integrate a PH culture [35] into the curriculum. As this year should encourage the establishment of ambulatory GP, it will also be an opportunity for these future doctors to think about a more sustainable practice, location, and organization of care. Ambulatory coordinated practice structures did not exist in France before 2007. In 2023, there were 2251 multiprofessional health houses, 455 multiprofessional health centers, and 389 territorial professional health communities [36,37]. GPs trained in PH and integrating them into health care systems could launch ambitious and original projects based on these sustainable structures, benefiting both patients and the planet. The 2023 report from the Lancet Countdown in Health and Climate Change reminded us that climate inaction is already costing lives and livelihoods today [38].

## Appendix

Multimedia Appendix 1 Study pretest and posttest questionnaires.

Multimedia Appendix 2 CONSORT (Consolidated Standards of Reporting Trials) 2010 checklist of information to include when reporting a pilot or feasibility trial.

## Abbreviations

CONSORT: Consolidated Standards of Reporting Trials
EH: environmental health
GP: general practitioner
PCEH: Primary Care Environment and Health
PH: planetary health
SPES: Soins Primaires Environnement et Santé

## Data Availability

The data sets generated and analyzed during this study are available from the corresponding author upon reasonable request.
