# Supplementary material for: An e-Learning Course to Train General Practitioners in Planetary Health: Pilot Intervention Study
Source: JMIR Form Res. 2024 May 14;8:e56138. doi: 10.2196/56138 (PMC11134242; doi:10.2196/56138)
Supplement: Multimedia Appendix 1 [file formative_v8i1e56138_app1.docx]

**Appendix 1**

**Pre-test questionnaire**

Hello,

You are about to follow a course module on planetary health in general practice,

accompanied by a questionnaire to be completed before and after the course. This

questionnaire is the subject of a medical thesis and also provides essential feedback on

the training module, so please fill it in carefully.

First part:

1) What is your gender (Male/Female)

2) What is your age (drop-down menu 15-50)

3) Have you taken part in any training courses on sustainable development/eco-responsibility during your time at university?

4) Are you concerned about climate change?

5) Do you already think you are taking eco-responsible action on a daily basis?

Second part :

1) Climate change is mainly due to human activity (Strongly disagree/Somewhat disagree/No agreement/Somewhat agree/Strongly agree)

2) Climate change is one of the major threats to human health. (Strongly disagree/Somewhat disagree/Not agree or disagree/Somewhat agree/Strongly agree)

3) By 2050, London could have the same climate as Barcelona around the year 2000. (Strongly disagree/Somewhat disagree/Neither agree nor disagree/Somewhat agree/Strongly agree)

4) The IPCC (Intergovernmental Panel on Climate Change) is a European group that has been in existence since 2000. (Strongly disagree/Somewhat disagree/Neither agree nor disagree/Somewhat agree/Strongly agree)

5) According to the IPCC, a rise of +1.5° in average global surface temperature would significantly worsen the consequences of climate change. (Strongly disagree/Somewhat disagree/Not agree or disagree/Somewhat agree/Strongly agree)

6) Overall, the populations that have contributed least to global warming are also the most exposed to its harmful effects. (Strongly disagree/Somewhat disagree/Neither agree nor disagree/Somewhat agree/Strongly agree)

7) According to the SHIFT Project, if it were a country, the healthcare sector would be the 5th largest emitter of greenhouse gases in the world. (Strongly disagree/Somewhat disagree/Not agree or disagree/Somewhat agree/Strongly agree)

8) Global Health is committed to promoting the health of all living things, not just human health. (Strongly disagree/Somewhat disagree/Not at all agree/Somewhat agree/Strongly agree)

9) In 2022, there will be 10 planetary limits, 6 of which have already been exceeded in France. (Strongly disagree/Somewhat disagree/Not agree or disagree/Somewhat agree/Strongly agree)

10) An MSP (Maison de Santé Pluriprofessionnelle) is obliged to incorporate an eco-responsible approach into its healthcare project. (Strongly disagree/Somewhat disagree/Neither agree nor disagree/Somewhat agree/Strongly agree)

Third part:

1) I feel confident in explaining the links between climate change and human health. (Strongly disagree/Somewhat disagree/Not agree or disagree/Somewhat agree/Strongly agree)

2) I feel confident in listing 3 consequences of climate change on human health and the health of ecosystems. (Strongly disagree/Somewhat disagree/Neither agree nor disagree/Somewhat agree/Strongly agree)

3) I feel confident about integrating eco-responsible practices into my personal life. (Strongly disagree/Somewhat disagree/Not agree or disagree/Somewhat agree/Strongly agree)

4) I feel confident about integrating eco-responsible practices into my professional life. (Strongly disagree/Somewhat disagree/Not agree or disagree/Somewhat agree/Strongly agree)

5) I feel confident about convincing my university tutor/hospital referral doctor to commit to an eco-responsible approach. (Strongly disagree/Somewhat disagree/Neither agree nor disagree/Somewhat agree/Strongly agree)

**Post-test questionnaire**

1. Scale your general knowledge level about environmental health (from 1 Poor to 5 Excellent).

Scale your knowledge level for each of the following environmental health parameters (from 1 Poor to 5 Excellent):

1. Outdoor air quality.
2. Indoor air quality in buildings (homes, schools, offices)
3. Noise.
4. Soil quality.
5. Radon
6. Carbon Monoxide.
7. Bathing water quality.
8. Tap water quality.
9. Legionnaire's disease
10. Endocrine disruptors.
11. Lead.
12. Other heavy metals (cadmium, aluminium).
13. Electromagnetic waves.
14. Pesticides.
15. Nanomaterials.
16. Allergenic plant pollens.
17. Vector-borne diseases (Chikungunya, Zika, yellow fever, malaria, etc).
18. Substandard housing.
19. The first 1000 days of life concept.
20. Web reporting of an environmental health risk.
21. The website signalement.social-sante.gouv.fr/ allows to report events at risk of environmental health, such as drugs sides effects reporting website (pharmacovigilance).

- Yes

- No

- I don’t know

1. Would you like to work on an environmental health thesis?

- Yes

- No

- I don’t know

1. Please write down here if you have ideas for a thesis topic in environmental health (free text).
2. After following the SPES training, scale how much you consider the possible effects of the environment on your health (consumption, protection, vigilance)? (From 1 Not at all to 5 Totally)
3. After following the SPES training, scale how much you consider the possible effects of the environment on your patients' health (consumption, protection, vigilance) (from 1 not at all to 5 totally).
4. Concerning the E-learning of the SPES training program, please rate your satisfaction level with the e-learning (only one answer possible):

-Completely satisfied

-Somewhat satisfied

-Don't know.

-Somewhat not satisfied

-Completely not satisfied

1. General comments on E-learning (free text)
2. Any comments on modules I, II, III, IV?

Regarding the different modules of the Primary Health Care and Environment (PHCE) training, please rate your level of satisfaction (from 1 poor to 5 Excellent) :

1. Module I Introduction
2. Module II Population approach
3. Module III Clinical cases
4. Module IV Communication.
5. Do you have any general comments/suggestions for the SPES training (free text)
